# Reporting of abstracts in studies that used routinely collected data for exploring drug treatment effects: a cross-sectional survey

**DOI:** 10.1186/s12874-021-01482-9

**Published:** 2022-01-07

**Authors:** Mei Liu, Wen Wang, Mingqi Wang, Qiao He, Ling Li, Guowei Li, Lin He, Kang Zou, Xin Sun

**Affiliations:** 1 Chinese Evidence-based Medicine Center and Cochrane China Center, National Clinical Research Center for Geriatrics, West China Hospital, Sichuan University, Chengdu, 610041 Sichuan China; 2 NMPA Key Laboratory for Real World Data Research and Evaluation in Hainan, Chengdu, China; 3 Sichuan Center of Technology Innovation for Real World Data, Chengdu, China; 4 Department of Health Research Methods, Evidence and Impact, McMaster University, Hamilton, ON L8S 4L8 Canada; 5 Center for Clinical Epidemiology and Methodology, Guangdong Second Provincial General Hospital, Guangzhou, 510317 Guangdong China; 6 Biostatistics Unit, Research Institute at St. Joseph’s Healthcare Hamilton, Hamilton, ON L8N 4A6 Canada; 7 Intelligence Library Center, West China Hospital, Sichuan University, Chengdu, 610041 Sichuan China

**Keywords:** Routinely collected data, Drug treatment effect, Cross-sectional study, Abstract reporting

## Abstract

**Background:**

In recent years, studies that used routinely collected data (RCD), such as electronic medical records and administrative claims, for exploring drug treatment effects, including effectiveness and safety, have been increasingly published. Abstracts of such studies represent a highly attended source for busy clinicians or policy-makers, and are important for indexing by literature database. If less clearly presented, they may mislead decisions or indexing. We thus conducted a cross-sectional survey to systematically examine how the abstracts of such studies were reported.

**Methods:**

We searched PubMed to identify all observational studies published in 2018 that used RCD for assessing drug treatment effects. Teams of methods-trained collected data from eligible studies using pilot-tested, standardized forms that were developed and expanded from “The reporting of studies conducted using observational routinely collected health data statement for pharmacoepidemiology” (RECORD-PE) statement. We used descriptive analyses to examine how authors reported data source, study design, data analysis, and interpretation of findings.

**Results:**

A total of 222 studies were included, of which 118 (53.2%) reported type of database used, 17 (7.7%) clearly reported database linkage, and 140 (63.1%) reported coverage of data source. Only 44 (19.8%) studies stated a predefined hypothesis, 127 (57.2%) reported study design, 140 (63.1%) reported statistical models used, 142 (77.6%) reported adjusted estimates, 33 (14.9%) mentioned sensitivity analyses, and 39 (17.6%) made a strong claim about treatment effect. Studies published in top 5 general medicine journals were more likely to report the name of data source (94.7% vs. 67.0%) and study design (100% vs. 53.2%) than those in other journals.

**Conclusions:**

The under-reporting of key methodological features in abstracts of RCD studies was common, which would substantially compromise the indexing of this type of literature and prevent the effective use of study findings. Substantial efforts to improve the reporting of abstracts in these studies are highly warranted.

**Supplementary Information:**

The online version contains supplementary material available at 10.1186/s12874-021-01482-9.

## Background

In recent years, routinely collected health data (RCD), such as electronic healthcare records and administrative claims, have been commonly used for exploring drug treatment effects [1–3]. However, such studies are often complex, not only in the use of data (e.g., epidemiology design and statistical analysis) but also reporting of methodological details and study findings. To enhance transparent reporting of studies using RCD, the REporting of studies Conducted using Observational Routinely collected health Data (RECORD) statement was issued in 2015 [4]. Subsequently, its extension to pharmacoepidemiology (RECORD-PE) was released [5].

In the reporting of studies using RCD, abstracts are often the first and probably the primary piece of information for clinicians and policy makers to read, which could have profound impact on the subsequent use of evidence by clinicians and policy makers [4]. Sufficient reporting would improve the assessment of study validity and results, and facilitate appropriate interpretation of study findings, thus achieving judicious use of the evidence [6–8]. One additional issue about such studies is how the information about RCD databases can be effectively indexed by literature databases (e.g., PubMed) to facilitate searching; this issue has been ignored and largely compromised identification of such studies.

Nevertheless, abstracts usually have strict word limits in the vast majority of journals, which often makes it highly challenging to adequately present important details. This is particularly the case for the studies using RCD, since such studies are inherently more complex in the methodological details and resulting findings than the classical well-established randomized controlled trials. Earlier studies found that the reporting of abstracts was often suboptimal [7, 9–11]. For instance, a survey of 124 studies using RCD—published in 2012—found that 62.9% of studies did not clearly describe study design in the abstract [11]. Another survey involving 25 studies suggested that only 44.0% of studies clearly reported data source [7]. However, these studies either included a relatively small sample size, were outdated, or did not focus on studies about drug effects. In addition, several important issues, such as how the investigators claimed treatment effect [12, 13], were not investigated before. Therefore, we conducted a cross-sectional survey of published RCD studies exploring drug treatment effects to investigate their reporting of titles or abstracts.

## Methods

### Eligibility criteria

We included studies that explicitly used RCD and a comparative study design (e.g., cohort study or case-control study) to explore drug treatment effects, including effectiveness and/or safety. RCD were defined as those data generated form routine care without a priori research purposes, such as electronic medical records, administrative claims data or insurance data. We excluded a study if it was unable to determine whether RCD were used. We also excluded studies that involved primary data collection for a research purpose.

### Search strategy

This study is part of a major research project addressing reporting and methodological issues about studies using RCD. Our major research project was conceptualized in early 2019. We searched PubMed to identify studies using RCD to explore treatment effects, published in the year of 2018 (search date September 18, 2019). We used terms correlated to routinely collected data, including “administrative claims data”, “routinely collected data” and “datalink”. We also integrated the search strategy for “electronic health records”, which was developed by the National Library of Medicine [14] and was peer-reviewed by an information specialist [11, 15], into our search. The details of the search strategy are presented in Additional file 1. The search was restricted to English language.

### Sample size and selection process

This study is part of a major research project addressing methodological issues about studies using RCD. The sample size for the major project was calculated based on number of factors that were potentially associated with study quality, measured as a continuous variable. These factors were selected according to group discussion and previously published studies [16–18]. Seven characteristics with eleven categories were taken in to consideration as independent variables, including whether the journal endorses RECORD (yes vs. no), the type of journal (top 5 general medicine journals versus other journals), the source of funding (any funding from for-profit organizations vs. funding exclusively from government or nonprofit organizations vs. no funding/not reported), type of data sources (EMR/EHR vs. claims vs. both), sample size (≤1000 vs. 1000–5000 vs. ≥5000), the type of outcome (exclusively safety outcome vs. exclusively effectiveness outcome vs. both safety and effectiveness outcome) and significance of the primary outcome (yes vs. no). Twenty studies per category were planned to provide sufficient observations and to avoid overfitting, resulting in a sample of 220 [19].

We stratified journals into top 5 general medicine journals and other journals according to impact factor (2018) from the Institute for Scientific Information (ISI) Web of Knowledge Journal Citation Reports. According to the impact factor of 2018, the top 5 general medicine journals included New England Journal of Medicine (NEJM), The Lancet, Journal of the American Medical Association (JAMA), British Medical Journal (BMJ), and JAMA Internal Medicine. We included all studies published in top 5 general medicine journals. With regard to studies published in other journals, we randomly sampled 1000 of the searched reports at a time and screened their tittles, abstracts and full texts for eligibility. We repeated the random sampling process until reaching the planned sample size of 220.

Two teams of paired, method-trained investigators (ML, WW, QH, MW) performed title/abstract screening in duplicate and independently. Subsequently, all potentially eligible full texts were screened by the two teams independently. We designed a Microsoft Access database, in which the screening forms and citation list were compiled, to perform the study screening. Decisions on inclusion or exclusion were entered into this database by investigators. Discrepancies were addressed through discussion or adjudication by a third reviewer (XS).

### Information extraction

We evaluated the reporting of titles or abstracts based on the RECORD-PE [5] checklist. The RECORD-PE checklist contained five items for titles and abstracts reporting, including the use of common study design terms, an informative and balanced summary about the research, the data source types and names, the linkage between databases, the geographical region and the timeframe. On the basis of the checklist, we developed structured, pilot-tested data extraction form to document whether the following items were reported: name of database; explicit statement of data source (i.e., healthcare, administrative, insurance, claims, primary care, secondary care, hospital); data coverage (i.e., single center, multiple or regional center, national center of international center); geographic region where the data came from; number of participants; follow-up duration; statistical methods (e.g., cox proportional hazard model); effect estimates (e.g., absolute risk, relative risk, confidence intervals or *P*-values, crude or adjusted estimates); mention of sensitive analysis; and mention of subgroup analysis.

In addition, we added new items deemed important specifically for RCD studies, through reviewing existing guidance documents [20–23] and brainstorming. We convened a group of five experts in pharmacoepidemiology, routinely collected health data research, and clinical epidemiology, to consult the importance and appropriateness of these items. Three items were finally included after multiple teleconferences meetings, including 1) whether specific wording was used to indicate the direction of effect, 2) whether a new user design was mainly considered, and 3) the claim about treatment effect (i.e., strong, moderate, weak). Using a pre-specified rule, we judged the strength of claim according to the statement authors made about the primary outcome in the conclusion of an abstract [24].

To ensure the quality of study screening and data abstraction, the data collection forms were pilot-tested and standardized. A sample of 20 studies were extracted and assessed by reviewers (ML, WW, QH, MW) to test the operationalization of the items and improve detailed extraction instructions. For the challenging item, such as claim of effect, 10% of the included full texts were randomly sampled for calibration exercise to ensure the consistency among reviewers. After calibration exercises, we achieved a good inner-reviewer agreement (kappa > 0.8).

### Data analysis

Descriptive analyses were conducted to evaluate the study characteristics of the included studies. We used descriptive analysis to explore the reporting characteristics of the data source, study conduction (including design, analysis and results) and interpretation in the title or abstracts. Categorical variables were presented as numbers and percentages. We also examined whether these characteristics differed in the journal impact (top 5 general medicine journals versus other journals).

Continuous variables were presented as the mean and standard deviation (SD) when normally distributed or otherwise the median and interquartile range (IQR). We used Stata/SE (version 14.0) for data analysis.

## Results

**Fig. 1:**
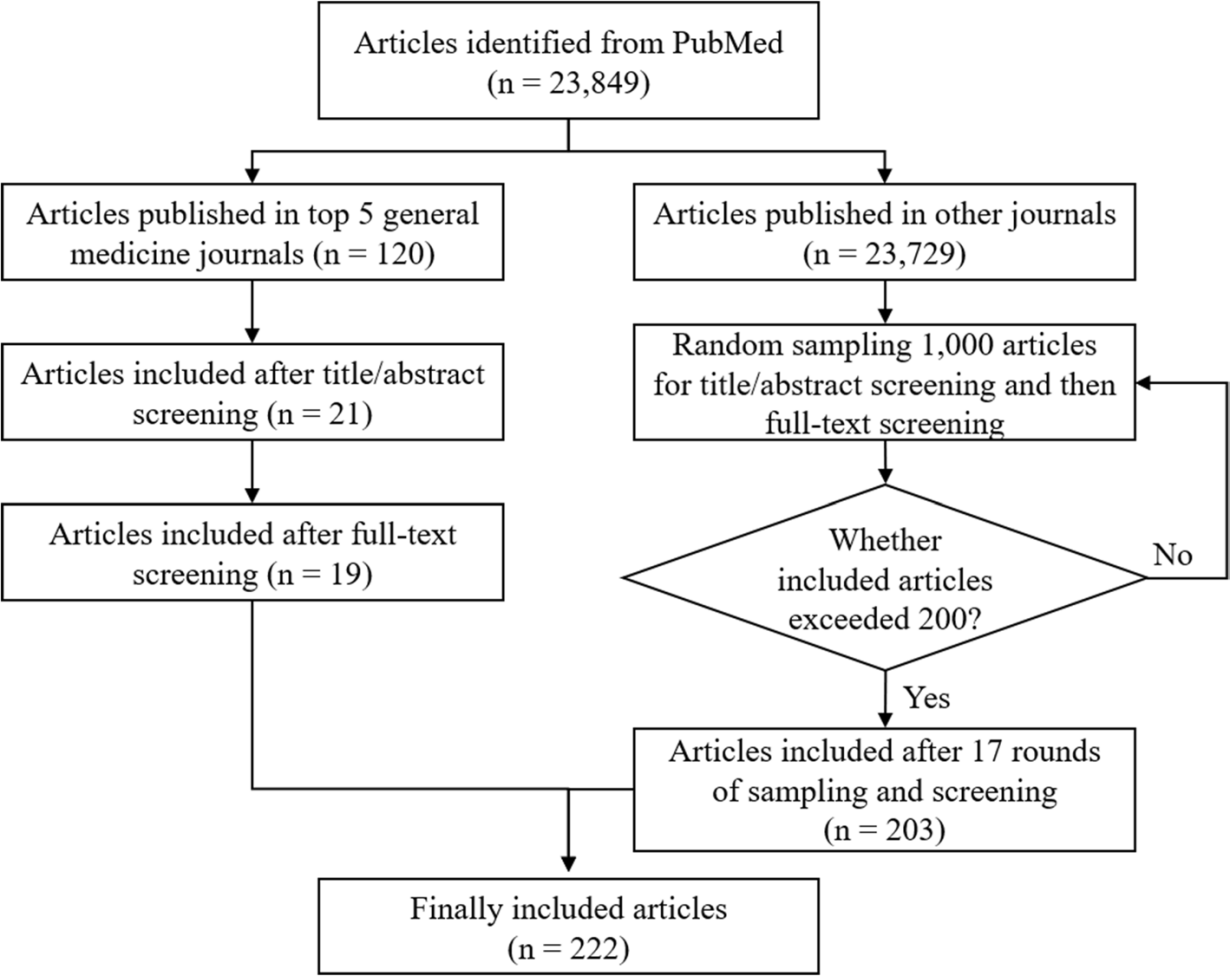
Flowchart of identification, screening and inclusion of studies

Our search yielded 23,849 reports. After full-text screening, 19 studies published in top 5 general medicine journals and 203 studies in other journals were finally included, a total of 222 studies (Fig. 1). Of the included studies, 35 (15.8%) were published in journals endorsing the RECORD Statement and 6 (2.7%) reported that they endorsed the RECORD Statement. Among included studies, 40 (18.02%) involved patients with endocrinologic disease, 40 (18.02%) involved patients with cardiovascular disease, 18 (8.11%) involved patients with cancers and 14 (6.31%) involved patients with mental health conditions. The general characteristics were displayed in Supplementary Table 1.

### Reporting of data source

Of the included studies, 17 (7.7%) clearly specified that they applied data linkage, and 118 (53.2%) reported type of databases used (Table 1); 154 (69.4%) reported name of database. However, 57 studies (25.7%) did not report any information about type of data source. Database coverage was reported in 140 (63.1%) studies, of which 72 (51.4%) used national data sources and 54 (38.6%) studies used multiple or regional data sources; 152 (68.5%) studies reported the country of origin, of which 46 (30.3%) studies used data sources from Taiwan, China, 34 (22.4%) from the US, and 21 (13.8%) from the UK.

**Table 1:** Reporting characteristics of the study data sources in the titles or abstracts of the included studies

Reporting item	Total	Journal type	
	(***n*** = 222)	Top 5 general medicine (***n*** = 19)	Other journals (***n*** = 203)
**Linkage between data sources,** ***n*** **(%)**	17 (7.7)	2 (10.5)	15 (7.4)
**Type of data source,** ***n*** **(%)**	118 (53.2)	10 (52.6)	108 (53.2)
**Categories of specific types,** ***n*** **(%)**			
EMR	38 (17.1)	6 (31.6)	32 (15.8)
Claims	53 (23.9)	3 (15.8)	50 (24.6)
RCD (not specified)^a^	21 (9.5)	2 (10.5)	19 (9.4)
Other^b^	17 (7.7)	0 (0.0)	17 (8.4)
**Where the data source type was reported,** ***n*** **(%)**			
Title	4 (3.4)	0 (0.0)	4 (3.7)
Abstract	98 (83.5)	10 (100.0)	88 (81.8)
Both the title and abstract	16 (13.6)	0 (0.0)	16 (14.8)
**Name of database,** ***n*** **(%)**	154 (69.4)	18 (94.7)	136 (67.0)
**Where the database name was reported,** ***n*** **(%)**			
Reported in the Title	1 (1.7)	0 (0.00)	1 (0.7)
Reported in the Abstract	146 (94.8)	18 (100.0)	128 (94.1)
Reported both in the Title and Abstract	7 (4.6)	0 (0.00)	7 (5.2)
**Name of database reported,** ***n*** **(%)**			
Full name	119 (77.3)	17 (94.4)	102 (75.0)
Abbreviation name	9 (5.8)	0 (0.0)	9 (6.6)
Both full and Abbreviation name	26 (16.9)	1 (5.6)	25 (18.4)
**Whether the name of database include data source type,** ***n*** **(%)**			
Yes	72 (46.8)	4 (22.2)	68 (50.0)
Partly^c^	5 (3.3)	1 (5.6)	4 (2.9)
Unclear^d^	8 (5.2)	1 (5.6)	7 (5.2)
Not include	69 (44.8)	12 (66.7)	57 (41.9)
**Coverage of data source,** ***n*** **(%)**	140 (63.1)	13 (68.4)	127 (62.6)
**Categories of specific coverage,** ***n*** **(%)**			
Single center	12 (8.6)	0 (0.0)	12 (9.5)
Multiple or regional center	54 (38.6)	3 (23.1)	51 (40.2)
National center	72 (51.4)	10 (76.9)	62 (48.8)
International center	2 (1.4)	0 (0.0)	2 (1.6)
**Country of data source,** ***n*** **(%)**	152 (68.5)	16 (84.2)	136 (67.0)
**Categories of Specific country**^e^**,** ***n*** **(%)**			
China, Taiwan	46 (30.3)	1 (6.3)	45 (33.1)
USA	34 (22.4)	3 (18.8)	31 (22.8)
UK	21 (13.8)	8 (50.0)	13 (9.6)
Korea	11 (7.2)	0 (0.0)	11 (8.1)
Japan	10 (6.6)	0 (0.0)	10 (7.4)

^a^ Administrative databases, healthcare databases, routinely collected databases were included

^b^ Birth registry and death registry were included

^c^ More than one name of data source was reported in one article, while not all names contained information that can indicate the type of data source (e.g., healthcare, claims, administrative, primary care or secondary care)

^d^ The name of the data source containing wording such as “clinical practice”, “health”, and “health information” was considered unclear as to whether they reflected the type of data source

^e^ Two studies used international data sources, one of which used data sources from the USA and three European countries. Another study used data sources from six European countries. Only top five items were list

### Reporting of study designs, statistical analyses and results

In total, 100 (45.1%) studies clearly reported the comparator in their conclusion; 127 (57.1%) studies reported study design, of which 96 (75.6%) were cohort study designs, 14 (11.0%) case-control designs and 16 (12.6%) nested case-controls; 57 (25.7%) reported the use of new user design, and 79 (35.6%) reported follow-up duration; 140 (63.1%) reported statistical models used.

A total of 113 (50.90%) studies reported absolute risk, of which 48 (42.5%) reported crude absolute risk and 9 (8.0%) reported adjusted absolute risk. Among 183 (82.4%) studies that reported relative risk, 142 (77.6%) were adjusted; 33 (14.9%) reported sensitivity analyses, in which 28 (84.9%) reported no significant change of findings; 33 (14.9%) reported subgroup analyses (Table 2).

**Table 2:** Reporting characteristics of the study design and analysis of the titles or abstracts of the included studies

Reporting item	Total	Journal type	
	(***n*** = 222)	Top 5 general medicine (***n*** = 19)	Other journals (***n*** = 203)
**Objective in a framed manner,** ***n*** **(%)**			
Population	186 (83.8)	14 (73.7)	172 (84.7)
Exposure	206 (92.8)	18 (94.7)	188 (92.6)
Comparator	99 (44.6)	12 (63.2)	87 (42.9)
Outcomes	203 (91.4)	17 (89.5)	186 (91.6)
**Study design,** ***n*** **(%)**	127 (57.2)	19 (100.0)	108 (53.2)
**Location of study design reporting**			
Reporting in title	21 (16.5)	1 (5.3)	20 (18.5)
Reporting in abstract	50 (39.4)	5 (26.3)	45 (41.7)
Reporting both in title and abstract	56 (44.1)	13 (68.4)	43 (39.8)
**Type of study design**			
Cohort study	96 (75.6)	17 (89.5)	79 (73.2)
Case-control	14 (11.0)	1 (5.3)	13 (12.0)
Nested case-control	16 (12.6)	1 (5.3)	15 (13.9)
Case-crossover	1 (0.8)	0 (0.0)	1 (0.9)
**Application of new user design,** ***n*** **(%)**	57 (25.7)	16 (84.2)	41 (20.2)
**Timeframe of research** ***n*** **(%)**	173 (77.9)	19 (100.0)	154 (75.9)
**Follow-up duration**	79 (35.6)	15 (79.0)	64 (31.5)
**Statistical model used**	140 (63.1)	9 (47.4)	131 (64.5)
**Categories of statistical model**			
Cox proportional hazard model	78 (55.7)	5 (55.6)	73 (55.7)
Logistic regression	34 (24.3)	2 (22.2)	32 (24.4)
Other ^a^	28 (20.0)	2 (22.2)	26 (19.9)
**Absolute risk** ^b,c^	113 (50.9)	10 (52.6)	103 (50.7)
Crude	48 (42.5)	5 (50.0)	43 (41.8)
Adjusted ^d^	9 (8.0)	2 (20.0)	7 (6.8)
Both	4 (3.5)	0 (0.0)	4 (3.9)
Not reported or unclear	52 (46.0)	3 (30.0)	49 (47.6)
**Relative risk** ^e^	183 (82.4)	17 (89.5)	166 (81.8)
Crude	2 (1.1)	0 (0.0)	2 (1.2)
Adjusted ^d^	136 (74.3)	14 (82.4)	122 (73.5)
Both	6 (3.3)	1 (5.9)	5 (3.0)
Not reported or unclear	39 (21.3)	2 (11.8)	37 (22.3)
**Estimators of relative risk** ^**g**^			
Risk ratio	11 (5.0)	1 (5.3)	10 (4.9)
Odds ratio	52 (23.4)	4 (21.1)	48 (23.7)
Hazard ratio	121 (54.5)	12 (63.2)	109 (53.7)
Other relative risk ^f^	4 (1.8)	1 (5.3)	3 (1.5)
**Sensitive analysis**	33 (14.9)	7 (36.8)	26 (12.8)
**Robustness of sensitive analysis**	28 (84.9)	7 (100.0)	21 (80.8)
**Subgroup analysis**	33 (14.9)	5 (26.3)	28 (13.8)

^a^ The multivariable generalized linear models, linear regression models, regression models without specifications (e.g., multiple regression model, multivariable regression model), fixed, random or mixed effect models, and other ambiguous statements were included (e.g., “inverse probability weighting model with regression adjustment”, “competing risk models”)

^b^ The absolute risk means the probability or change that the outcome will occur, including the incidence rate, number of events or rate difference between exposure and comparator

^c^ Did the article reporting absolute risk clearly specify or indicate whether the absolute risk was crude or adjusted? Both crude and adjusted were considered “yes”, and not clearly specified was considered “no”

^d^ Statements such as “multivariate analysis”, “propensity score adjustment”, “adjusted some covariates” and claims of adjusted estimators were considered when determining that the reported absolute risk was adjusted

^e^ Relative risk refers to the chance that participants will experience events in the exposure group compared to the comparator group or the exposure rate in the case compared to the control group

^f^ Other relative risks mainly include incidence rate ratios (IRRs)

^g^ Did the article reporting relative risk clearly specify or indicate whether the relative risk was crude or adjusted? Both crude or adjusted were considered “yes”, and not clearly specified was considered “no”

### Interpretation of findings

Of the included studies, only 44 (19.8%) clearly stated a predefined hypothesis, and 61 studies (27.5%) yielded negative findings. Among studies with a predefined hypothesis, 8 (18.6%) reported inconsistent results with their prespecified hypotheses. In the reporting of primary outcome, 39 (17.6%) had a strong treatment effect claim, 143 (64.4%) conveyed a moderate claim and 40 (18.0%) conveyed a weak claim (Table 3).

**Table 3:** Interpretation characteristics of statistical analysis in title or abstract of the included article

Reporting item	Total	journal rank	
	(***n*** = 222)	Top 5 general medicine (***n*** = 19)	Other journals (***n*** = 203)
**Predefined hypothesis** ^a^**,** ***n*** **(%)**	44 (19.8)	6 (31.6)	38 (18.7)
**Conclusion in a framed manner, n (%)**			
Population	176 (79.3)	13 (68.4)	163 (80.3)
Exposure	206 (92.8)	16 (84.2)	190 (93.6)
Comparator	100 (45.1)	10 (52.6)	90 (44.3)
Outcomes	203 (91.4)	19 (100.0)	184 (90.6)
**Direction of the treatment effect**			
Positive	170 (76.6)	17 (89.5)	153 (75.4)
Negative	61 (27.5)	4 (21.1)	57 (28.1)
**Consistency with predefined hypothesis**			
Consistent	29 (67.4)	3 (50.0)	26 (70.3)
Not consistent	8 (18.6)	1 (16.7)	7 (18.9)
Partly consistent	6 (14.0)	2 (33.3)	4 (10.8)
**Claim of the treatment effect**			
Strong^b^	39 (17.6)	4 (21.1)	35 (17.2)
Moderate^c^	143 (64.4)	11 (57.9)	132 (65.0)
Weak^d^	40 (18.0)	4 (21.1)	36 (17.7)

^a^ Specific wording that indicates the direction of risk between exposure and outcome (e.g., increase, decrease) will be considered a predefined hypothesis

^b^ Authors convey a conviction that the treatment effect truly exists

^c^ Authors convey a belief that the treatment effect possibly exists

^d^ Authors suggest a treatment effect but convey uncertainty about whether such an effect exists

### Comparisons between top 5 general medicine and other journals

Studies published in top 5 general medicine journals were more likely to include more participants (median: 154,162 vs. 15,597), involve methodologists (79.0% vs. 56.2%), and receive nonprofit grants (84.2% vs. 48.3%) than those published in other journals (Supplementary Table 1).

Compared to studies in lower impact journals, those in top 5 general medicine journals were more likely to report name of database (94.7% vs. 67.0%), study design (100% vs. 53.2%) and follow-up time (79.0% vs. 31.5%), and apply a new user design (84.2% vs. 20.2%). Comparative rates were found in the reporting of type of data source (52.6% vs. 53.2%) and statistical models (47.4% vs. 64.5%).

## Discussions

### Main findings and interpretations

In recent years, studies that used RCD for exploring drug treatment effects have rapidly increased, thanks to the increasing availability of these data and the rapid development of complex epidemiological and statistical methods. However, its special methodological features—such as the use of existing healthcare databases, complex pharmacoepidemiological designs and statistical methods—often make these studies differ from traditional observational studies. Clear reporting of these methodological details in the abstracts have important implications for these studies to be effectively indexed in the literature database and to be appropriately used by clinicians and policy makers.

Our study found important limitations in the reporting of abstracts among studies that used RCD for exploring treatment effects. In identifying themselves as database studies, nearly half of the studies did not clearly specify type of data sources, and a quarter did not report any detailed information regarding data sources. In the reporting of important methodological features, we also found that only 57.2% of studies reported study designs, 63.1% reported statistical models, and 77.9% reported time frame of the research. Only 19.8% clearly reported predefined hypotheses.

We also found that reporting of abstracts was generally better among top 5 general medicine journals. However, such important items as type of data source and statistical models were not well reported across all journals.

Our study strongly suggested the need to improve the reporting of abstracts among studies using RCD to examine drug treatment effects. In particular, the reporting of database information is critical in identifying such studies as a database study. Both authors and journal editors may consider adding a suffix to the title and claiming them as database studies. In addition, authors should also consider concisely describe the type of database and the data source to enable better capture of key methodological features of the study.

Although abstracts are usually highly condensed, both authors and journal editors should consider include minimal requirements about the reporting of epidemiological designs and statistical methods and should always consider reporting adjusted effect estimates. Although only a relatively small proportion of studies made a strong claim of treatment effects in the abstracts, this may sometimes mislead decisions, as the nature of the abstract may not be able to convey sufficient information for readers to judge if the claim is appropriate. At least, authors should be highly cautious in the claim of treatment effect in the abstracts. In summary, existing reporting guidelines— such as RECORD and RECORD-PE—are important to improve reporting of these studies [4, 5, 17]. Authors and journal editors should work together to adhere to these requirements.

### Comparison with previous studies

Similar to our findings, previously published studies showed substantial reporting deficits in several important items among RCD studies [7, 11], including research questions, types of data sources, geographic regions and time frames, study designs, and statistical models. For instance, a study including 25 RCD studies showed that only 44.0% of studies mentioned type of database in abstract, and only 56.0% reported geographic region and time frame [7]. Another study also found that over a quarter of the studies did not contain clear wording regarding the type of data and 62.9% did not adequately report study design in the abstract [11]. Although a relatively high proportion of studies (69.4%) reported the name of the database in this study, naming the database cannot replace reporting the type of data source in articles indexing [25].

Our findings showed that top journals were associated with better general reporting, consistent with previous studies [11, 26]. For instance, a survey involving 124 RCD studies found a significant association between the journal impact factor and reporting domains such as statistical analyses, outcomes, and the coding and classification of participants [11]. Nevertheless, the reporting in some domains remains suboptimal regardless of impact factors [10, 11]. Only 47.3% of studies published in top-5 general medicine journals clearly reported a statistical model in our study, lower than that in other journals (64.5%).

### Strengths and limitations

There are several strengths in our study. First, we included the identification of large number of representative RCD studies exploring drug treatment effects by systematically researching. Second, we used standardized and pilot-tested forms for information extraction, and calibration exercises for reviewers to improve the extraction accuracy.

Our study has some limitations. First, there was a lack of accepted MeSH subject headings for RCD. By using search terms related to RCD, we may have missed studies. Nevertheless, the search strategy was developed together with information specialists, which aimed to ensure completeness of searching. Second, our sample was drawn from English publications in PubMed-indexed journals in 2018, and only 19 studies published in top 5 general medical journals were included. Therefore, our findings may not be generalizable to other years and journals outside the sample. They also may not be representative of all papers published in top 5 medical journals. Nevertheless, the PubMed database included a wide spectrum of journals. Moreover, the reporting of such studies was unlikely to have a significant change in a relatively short time [11]. Third, the judgment on some of the items may be subject to change across investigators. To ensure consistency of the judgement, we developed detailed instructions for these items and performed calibration exercises.

## Conclusion

Our work revealed that under-reporting of important methodological characteristics was common in abstracts of studies that used RCD for exploring drug treatment effects. Clear reporting of research hypotheses, information about database, epidemiological designs, statistical methods, and key findings is critical. In addition, authors should also be cautious in the claim of treatment effects in an abstract, since the findings from the studies using RCD may often be susceptible to bias.

## Supplementary Information

**Additional file 1.** Search Strategies.

**Additional file 2: Supplementary Table 1.** Basic characteristics of the analyzed electronic healthcare data study sample.

## Data Availability

The datasets analyzed during the current study available from the corresponding author on reasonable request.

## Abbreviations

RCD: Routinely Collected Data
RECORD: REporting of studies Conducted using Observational Routinely collected health Data
RECORD-PE: the RECORD for Pharmacoepeidemiology
EMR: Electronic Medical Record
EHR: Electronic Health Record
ISI: Institute for Scientific Information
NEJM: New England Journal of Medicine
JAMA: Journal of the American Medical Association
BMJ: British Medical Journal
