# Supplementary material for: Reporting of abstracts in studies that used routinely collected data for exploring drug treatment effects: a cross-sectional survey
Source: BMC Med Res Methodol. 2022 Jan 7;22:6. doi: 10.1186/s12874-021-01482-9 (PMC8742367; doi:10.1186/s12874-021-01482-9)
Supplement: Supplementary file 1 — Additional file 1. Search Strategies. [file 12874_2021_1482_MOESM1_ESM.docx]

Additional file 1 Search Strategy

1. ("Databases as Topic"[mh] OR database*[tiab] OR "health care databases"[tiab] OR "healthcare databases"[tiab] OR "health care database"[tiab] OR "healthcare database"[tiab] OR "healthcare data"[tiab] OR "health care data"[tiab] OR "national database"[tiab])
2. ((health information exchange[tw] OR hie[tw] OR rhio[tw] OR regional health information organization[tw] OR hl7[tw] OR health level seven[tw] OR "unified medical language system"[MeSH Major Topic] OR umls[tw] OR loinc[tw] OR rxnorm[tw] OR snomed[tw] OR icd9 cm[ti] OR icd 9 cm[ti] OR icd10[ti] OR icd 10[ti] OR metathesaurus[tw] OR patient card[tw] OR patient cards[tw] OR health card[tw] OR health cards[tw] OR electronic health data[tw] OR personal health data[tw] OR personal health record[tw] OR personal health records[tw] OR "health records, personal"[MeSH Major Topic] OR "health records, personal"[MeSH Major Topic] OR ehealth[tw] OR e-health[tw] OR "medical informatics applications"[MeSH Terms] OR "medical informatics applications"[MeSH Terms] OR "medical records systems, computerized"[MeSH Terms] OR "medical records systems, computerized"[MeSH Terms] OR computerized patient medical records[tw] OR automated medical record system[tw] OR automated medical record systems[tw] OR automated medical records system[tw] OR automated medical records systems[tw] OR computerized medical record[tw] OR computerized medical records[tw] OR computerized patient records[tw] OR computerized patient record[tw] OR computerized patient medical record[tw] OR electronic health record[tw] OR electronic health records[tw] OR "electronic health records"[MeSH Major Topic] OR "electronic health records"[MeSH Major Topic] OR electronic patient record[tw] OR electronic patient records[tw] OR electronic medical record[tw] OR electronic medical records[tw] OR electronic healthcare records[tw] OR electronic healthcare record[tw] OR electronic health care record[tw] OR electronic health care records[tw] OR "archives"[MeSH Major Topic] OR ehr[tw] OR ehrs[tw] OR phr[tw] OR phrs[tw] OR emr[tw] OR emr[tw] OR "health information systems"[MeSH Major Topic] OR "health information systems"[MeSH Major Topic] OR "health information systems"[MeSH Major Topic] OR "health information interoperability"[MeSH] OR "health information interoperability"[tw]) AND (medical record[ti] OR "medical records"[MeSH Terms] OR medical records[ti] OR patient record[ti] OR patient records[ti] OR patient health record[ti] OR patient health records[ti] OR "patient identification systems"[MeSH Terms] OR "patient identification systems"[MeSH Terms] OR "patient outcome assessment"[MeSH Major Topic] OR "patient discharge summaries"[MeSH Major Topic] OR healthcare record[ti] OR healthcare records[ti] OR health care record[ti] OR health care records[ti] OR health record[ti] OR health records[ti] OR hospital information system[tw] OR hospital information systems[tw] OR umae[ti] OR "attitude to computers"[MeSH Terms] OR medical informatics[ti]OR "information technology"[MeSH] OR "information technology"[tw])) OR (("medical records systems, computerized"[MeSH Major Topic] OR "medical records systems, computerized"[MeSH Terms] OR computerized patient medical record[tw] OR computerized patient medical records[tw] OR automated medical record system[tw] OR automated medical record systems[tw] OR automated medical records system[tw] OR automated medical records systems[tw] OR computerized medical record[tw] OR computerized medical records[tw] OR computerized patient records[tw] OR computerized patient record[tw] OR "patient generated health data"[mesh] OR "patient generated health data"[tw]OR electronic health record[tw] OR electronic health records[tw] OR electronic patient record[tw] OR electronic patient records[tw] OR electronic medical record[tw] OR electronic medical records[tw] OR electronic healthcare records[tw] OR electronic healthcare record[tw] OR electronic health care record[tw] OR electronic health care records[tw] OR "unified medical language system"[MeSH Major Topic] OR unified medical language system[tw] OR umls[tw] OR loinc[tw] OR rxnorm[tw] OR snomed[tw] OR icd9 cm[ti] OR icd 9 cm[ti] OR icd10[ti] OR icd 10[ti] OR Metathesaurus[tw] OR ehr[tw] OR ehrs[tw] OR phr[tw] OR phrs[tw] OR emr[tw] OR emrs[tw] OR meaningful use[tiab] OR meaningful use[tw] OR "meaningful use"[MeSH Major Topic]) AND ("J AHIMA"[Journal] OR "J Am Med Inform Assoc"[Journal] OR "AMIA Annu Symp Proc"[Journal] OR "Health Data Manag"[Journal] OR "Int J Med Inform"[Journal] OR "Yearb Med Inform"[Journal] OR "Telemed J E Health"[Journal] OR "Stud Health Technol Inform"[Journal]))
3. (Administrative[tiab] OR Claims[tiab] OR "routine data" [tiab] OR "routinely collected" [tiab])
4. ("retrospective database"[tiab] OR "secondary data"[tiab] OR "medical insurance" [tiab] OR "datalink"[tiab])
5. #1 OR #2 OR #3 OR #4
6. "humans"[mh]
7. English[lang]
8. #5 AND #6 AND #7
9. ((Review[ptyp] OR Address[ptyp] OR Biography[ptyp] OR Bibliography[ptyp] OR Autobiography[ptyp] OR Case Reports[ptyp] OR Clinical Conference[ptyp] OR Comment[ptyp] OR Congress[ptyp] OR Consensus Development Conference[ptyp] OR Consensus Development Conference, NIH[ptyp] OR Editorial[ptyp] OR Letter[ptyp] OR Dictionary[ptyp] OR Directory[ptyp] OR Historical Article[ptyp] OR Legal Case[ptyp] OR Meta-Analysis[ptyp] OR Guideline[ptyp] OR News[ptyp] OR Newspaper Article[ptyp] OR Patient Education Handout[ptyp] OR Personal Narrative[ptyp] OR Practice Guideline[ptyp] OR Interview[ptyp] OR Legislation[ptyp] OR Lecture[ptyp] OR Video-Audio Media[ptyp] OR Webcasts[ptyp] OR Portrait[ptyp]))
10. #8 NOT #9
11. Filters: Publication date from 2018/01/01 to 2018/12/31
