# Supplementary material for: Reporting of abstracts in studies that used routinely collected data for exploring drug treatment effects: a cross-sectional survey
Source: BMC Med Res Methodol. 2022 Jan 7;22:6. doi: 10.1186/s12874-021-01482-9 (PMC8742367; doi:10.1186/s12874-021-01482-9)
Supplement: Supplementary file 2 — Additional file 2: Supplementary Table 1. Basic characteristics of the analyzed electronic healthcare data study sample. [file 12874_2021_1482_MOESM2_ESM.docx]

**Supplementary Table 1 Basic characteristics of the included studies**

| **Characteristics** | **Total** | **journal type** | |
| --- | --- | --- | --- |
|  | **(n = 222)** | **Top 5 general medicine (n=19)** | **Other journals (n=203)** |
| **Number of participants, median (IQR)** | 17961 (2495, 92366) | 154162 (58994, 289469) | 15597 (1925, 80198) |
| ≤ 1,000 | 28 (12.61) | 0 (0.00) | 28 (13.79) |
| 1,000 – 5,000 | 43 (19.37) | 1 (5.26) | 42 (20.69) |
| ≥ 5,000 | 151 (68.02) | 18（94.74） | 133 (65.52) |
| **Participant diseases** ^a^**, *n* (%)** |  |  |  |
| endocrinologic | 40 (18.02) | 6 (31.58) | 34 (16.75) |
| cardiovascular | 40 (18.02) | 5 (26.32) | 35 (17.24) |
| cancers | 18 (8.11) | 0 (0.00) | 18 (8.87) |
| mental health conditions | 14 (6.31) | 1 (5.26） | 13 (6.40) |
| respiratory | 11 (4.95) | 1 (5.26) | 10 (4.93) |
| **Methodologist involvement** ^b^**, *n* (%)** |  |  |  |
| yes | 129 (58.11) | 15 (78.95) | 114 (56.16) |
| no | 62 (27.93） | 0 (0.00) | 62 (30.54) |
| Unclear ^c^ | 31 (13.96) | 4 (21.05) | 27 (13.30) |
| **Funding ^d^, *n* (%)** |  |  |  |
| not for profit | 114 (51.35) | 16 (84.21) | 98 (48.28) |
| private for profit | 41 (18.47) | 3 (15.79) | 38 (18.72) |
| not funded or not reported | 67 (30.18) | 0 (0.00) | 67 (33.00) |

a: Only the top five items were listed.

b: Methodologists include epidemiologists, statisticians and informatics.

c: The departments, such as medical research centers, health solutions, and health economics, were considered unclear when we judged whether there were epidemiologists or statisticians; departments, such as the Institute for Health Policy and Medical Science, or descriptions such as “somebody had full access to all the data…” were considered unclear when we judged whether there were informatics.

d: If funding included both not for profit and private for profit it was categorized as private for profit.
